# The role of health and social factors in education outcome: A record-linked electronic birth cohort analysis

**DOI:** 10.1371/journal.pone.0220771

**Published:** 2019-08-09

**Authors:** Annette Evans, Frank Dunstan, David L. Fone, Amrita Bandyopadhyay, Behnaz Schofield, Joanne C. Demmler, Muhammad A. Rahman, Ronan A. Lyons, Shantini Paranjothy

**Affiliations:** 1 Division of Population Medicine, School of Medicine, Cardiff University, Cardiff, Wales, United Kingdom; 2 Health Data Research UK, Swansea University, Wales, United Kingdom; Univesity of Iowa, UNITED STATES

## Abstract

**Background and objective:**

Health status in childhood is correlated with educational outcomes. Emergency hospital admissions during childhood are common but it is not known how these unplanned breaks from schooling impact on education outcomes. We hypothesised that children who had emergency hospital admissions had an increased risk of lower educational attainment, in addition to the increased risks associated with other health, social and school factors.

**Methods:**

This record-linked electronic birth cohort, included children born in Wales between 1 January 1998 and 31 August 2001. We fitted multilevel logistic regression models grouped by schools, to determine whether emergency hospital inpatient admission before age 7 years was associated with the educational outcome of not attaining the expected level in a teacher-based assessment at age 7 years (KS1). We adjusted for pregnancy, perinatal, socio-economic, neighbourhood, pupil mobility and school-level factors.

**Results:**

The cohort comprised 64 934 children. Overall, 4680 (7.2%) did not attain the expected educational level. Emergency admission to hospital was associated with poor educational attainment (OR 1.12 95% Credible Interval (CI) 1.05, 1.20 for all causes during childhood, OR 1.19 95%CI 1.07, 1.32 for injuries and external causes and OR 1.31 95%CI 1.04, 1.22 for admissions during infancy), after adjusting for known determinants of education outcomes such as extreme prematurity, being small for gestational age and socio-economic indicators, such as eligibility for free school meals.

**Conclusion:**

Emergency inpatient hospital admission during childhood, particularly during infancy or for injuries and external causes was associated with an increased risk of lower education attainment at age 7 years, in addition to the effects of pregnancy factors (gestational age, birthweight) and social deprivation. These findings support the need for injury prevention measures and additional support in school for affected children to help them to achieve their potential.

## Introduction

The determinants of educational outcomes in children are multifactorial, with complex interactions between various biological, social and environmental factors. Most previous research has either measured health status in children using a combination of chronic health conditions and any hospital admission [1–2] or looked at the impact of specific chronic conditions such as diabetes, sickle cell disease, cardiovascular disorders, asthma, ADHD, autism, seizure disorders, with conflicting results [3–5]. There is limited previous research on the effects of acute, transient conditions that lead to unplanned hospital admission on educational attainment, and previous published literature mainly look only at specific conditions. None of these studies have examined the combined contribution of other factors also known to be associated with education outcomes, such as health status at birth (for example, gestational age and birthweight) socio-economic and school level factors.

Health status at birth is important because lower than expected educational attainment in the early school years has been closely linked to birthweight and gestational age. Children born with low [6–7] and extremely low birthweight [8], or born before term [9–12], have a higher risk of lower educational attainment. The risk of not attaining academically appears to increase with each earlier week of gestation at birth [13–14], and each decrease in kilogram of birthweight (adjusted for gestation) [7]. These children are more likely to have poor long term cognitive and school outcomes across a range of measures [15], reduced language abilities [16], special educational needs [17], and higher rates of morbidity, including emergency hospital admissions during childhood [18].

Socio-economic and school level factors are also known important determinants of educational outcome. Multilevel research on educational progress has shown the importance of neighbourhood deprivation, pupil mobility, and school-level factors in explaining educational achievement [19–21]. Although moving schools and the learning environments created by teachers and schools may account for some of the unexplained variation in school performance, previous studies investigating the relationship between child health and educational attainment have not adjusted for the influences of these important exposures.

In this study we aimed to understand how health status measured at different stages in the early life course (pregnancy to age 7 years) is associated with educational outcome at age 7 years, in combination with the effects of socio-economic, neighbourhood, pupil mobility and school-level factors. We hypothesised that children who had emergency hospital admissions had an increased risk of lower educational attainment, over and above the increased risks associated with other health (e.g. pregnancy and perinatal) and social factors.

We used emergency hospital admissions as a measure of health status because they are common and occur at short notice as a result of clinical need, primarily because of respiratory and gastro-intestinal infections in younger children and due to injuries and external causes in older children [22]. We have already shown that pregnancy and perinatal factors such as maternal smoking during pregnancy, lower gestational age, low birthweight, and not breastfeeding is associated with increased rates of emergency admissions in children [23]. In this paper, we extend this analysis to look at how these pregnancy and perinatal factors, and emergency admissions during childhood collectively impact on education outcome. In order to understand how hospital admissions might impact on educational outcomes we examined the effect of amount of time spent in hospital, timing of admission (before or after starting school), age at first admission and cause of admission.

## Methods

### Data sources

We analysed data from the Wales Electronic Cohort for Children (WECC) [24]. WECC includes children born over a 19-year period between 01/01/1990 and 31/08/2008, with the following inclusion criteria: (1) born in Wales, or elsewhere with a mother usually resident in Wales, or (2) child born in the same 19-years period, resident in Wales from in-migration. Eligible children are identified from the Wales Demographic Service (WDS), a register of all individuals registered with a General Medical Practitioner and the National Community Child Health (NCCHD) Dataset, which is a register of all births in Wales. Each person is assigned an Anonymised Linking Field (ALF_E), which is used to link across multiple datasets. Child ALF_Es were linked with the mother’s ALF_E, allowing anonymised record linkage to maternal health records. The cohort is held in the Secure Anonymised Information Linkage (SAIL) databank at the Health Data Research UK in Swansea University [25–26]. Data on pregnancy and birth were linked from five databases in Wales as described in Table 1 and includes information on births, deaths, gestation, birthweight, and congenital anomalies.

**Table 1:** WECC pregnancy and birth characteristics.

Data source	Description
Public Health Birth files from the Office for National Statistics (from 2003)	Data on all births in Wales or to mothers who are usually resident in Wales
Public Health Mortality Files from the Office for National Statistics (from 2002)	Data on all deaths in Wales or of individuals who are usually resident in Wales
All Wales Perinatal Survey (from 1993)	A database of perinatal and infant mortality in Wales (including infants from 20 weeks’ gestation to 1 year of age), whose mother is usually resident in Wales or who die in a Welsh hospital
National Community Child Health Database (from 1987)	A national database of all children resident in Wales or born in a Welsh hospital, and contains data collected at birth including gestation, birth weight, and mode of delivery.
Congenital Anomaly Register and Information Service (from 1998)	A population-based register of any foetus or infant who has a congenital anomaly whose mother is usually resident in Wales at the time of birth; the definition of congenital anomalies is defined by the European network of population-based registries of epidemiologic surveillance of congenital anomalies

### Cohort dataset for analysis

**Fig 1:**
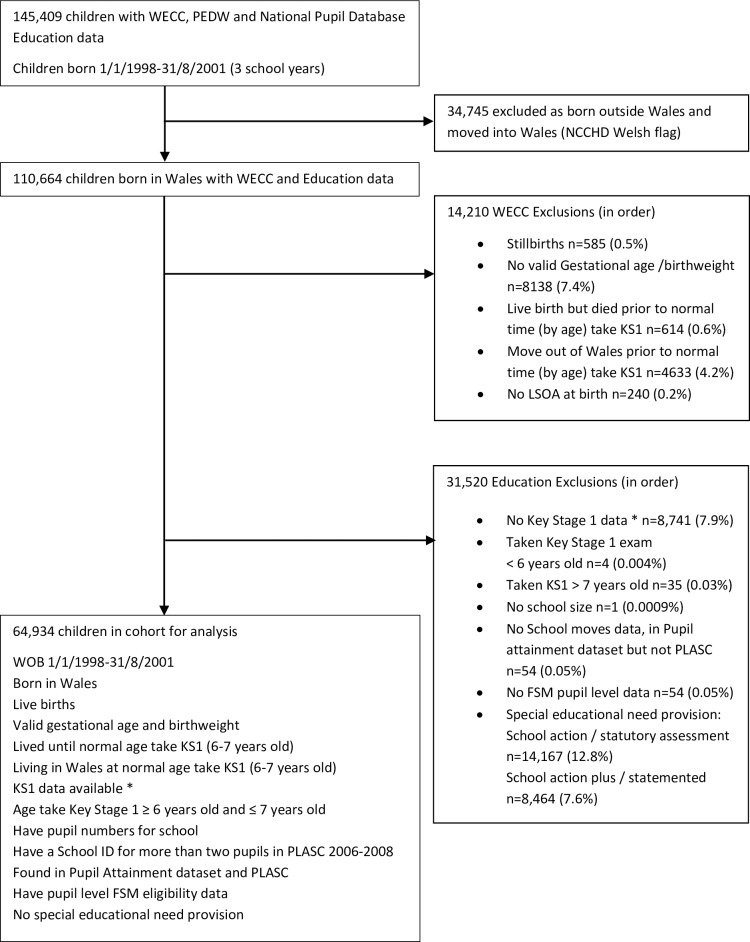
Anonymised participant selection for education and hospital admissions data within Wales Electronic Cohort for Children. WECC = Wales Electronic Cohort for Children, PEDW = Patient Episode Database Wales, NCCHD = National Community Child Health Database, KS1 = Key Stage 1, LSOA = Lower Super Output Area, PLASC = Pupil Level Annual School Census, FSM = Free school meal. *Children with no Key Stage 1 data do not attend schools maintained by the Local Education Authority or do not have a KS1 assessment (private schools, severely disabled children who do not enter the Special Educational Needs status system, those outside administrative systems e.g. Travellers).

For this study we used record-linked data on babies born in Wales between 1 January 1998 and 31 August 2001 as this cohort of children had both educational data recorded at age 7 years and linked hospital admission data available from birth. The cohort for analysis was 64 934 children with a Key Stage 1 (KS1) assessment between the three school years 2005/06 and 2007/08. Children born outside of Wales were excluded as these children do not have pregnancy or birth data available. The other main categories of exclusion were children who had moved out of Wales, children with Special Education Needs (SEN) provision, missing birthweight or gestational age, stillbirths and deaths, and missing educational data. The flow chart defining the cohort for analysis is shown in Fig 1.

### Outcome measure

The National Pupil Database (NPD) and Pupil Level Annual School Census (PLASC) database, that contain yearly educational attainment data for all pupils registered in schools in Wales maintained by their local education authority, were linked into the WECC dataset. Our outcome measure of educational attainment was the KS1 teacher-based assessment against attainment targets within the national curriculum of educational achievement in language, science and mathematics. Children were normally assessed in the school year (September 1st to August 31st) that included their 7^th^ birthday. Pupils in Welsh-speaking schools or classes were assessed by teacher assessment against the attainment targets for the Welsh language instead of English. Each child was awarded a level of achievement at KS1, usually between the lowest level 1 and level 3, where the expected level of attainment at KS1 is level 2.

The primary outcome in this study was a binary variable of whether the expected level (at least level 2 in all three KS1 assessments) was attained or not (less than level 2 in at least one assessment). Children who did not attain KS1 included those who were a) taught the KS1 curriculum but were not awarded at least level 2, b) unable to be assessed (usually due to physical difficulties), c) working towards level 2 (but had not completed enough of the curriculum to take the test) or d) disapplied (either due to special educational needs or a migrant currently unable to speak the language being tested). In 2008, approximately 20% of children in Wales who did not attain KS1were awarded level 1, 0.1% were not able to take assessment, 4.8% were working towards level and 0.2% were disapplied [27]. Our three secondary outcomes respectively recorded attaining at least level 2 in each of the three language, science and mathematics domains separately.

The education datasets included all children who attended a school maintained by the Local Education Authority (LEA). Children who did not have a KS1 assessment (and excluded from this analysis) included those attending independent schools (approximately 2% of children), severely disabled children and children with some major congenital anomalies (approximately 4.3% of children in Wales), those outside administrative systems, such as travellers, and children who took KS1 later than the normal time of age 6–7 years old. We also excluded children with SEN provision from the analyses as their chances of attaining a satisfactory level at KS1 were much lower than for other children.

### Hospital admissions

Data on emergency inpatient hospital admissions were linked from the Patient Episode Dataset for Wales (PEDW), which includes demographic and clinical data on beds used for daycases and inpatients to National Health Service (NHS) Wales hospitals and all Welsh residents treated in England from 1 January 1998. The emergency inpatient hospital admissions data used in this analysis included any unplanned hospital admission either through accident and emergency departments or a direct admission to a ward.

We extracted the International Classification of Disease 10^th^ Revision (ICD-10) [28] diagnosis code in the first, subsidiary and second fields of the 14 coding positions for each individual child’s emergency inpatient admissions. For each child we defined a binary variable recording if there had been any emergency inpatient admissions between birth and the 1^st^ of September (that is, the first day of school) in the school year in which they were assessed for KS1. We chose to use this date because work undertaken throughout the school year can be included in the teacher-based assessment of KS1, which usually takes place in the summer term. This enabled us to preserve the temporal order of exposure and outcome in the analyses. We also derived binary variables for the common causes of childhood emergency inpatient admissions. These were classed as respiratory, injury and poisoning, gastro-intestinal using the ICD-10 chapter headings J, S to Y and K respectively and ‘other’ including all other diagnosis codes. We coded the total number of all emergency inpatient admissions for each child and derived a second variable for the number of bed days, created by subtracting the date(s) of discharge from the date(s) of admission for each child. We report bed days in multiples of 10 bed days compared to no bed days for ease of interpretation. This variable was calculated for two time periods: from birth to the start of school, and from starting school to the start of the school year in which KS1 was assessed, so that we could examine whether or not the timing of emergency admissions had a differential effect on educational outcome. Finally we derived a variable for age at first admission with the categories of never admitted (reference), under 1 year of age (period during which neonatal conditions are the biggest contributor to morbidity), pre-school (between 1 year and 4 years of age on 1st September of the school entry year, when community acquired infections are more prevalent) and school-age (age 4+ years between school entry date and the start of the school year in which KS1 attainment was assessed, during which time injuries and external causes are a major contributor to morbidity). We chose these time periods because they reflect the different stages of the early life course.

### Individual-level variables

#### Pregnancy and birth covariates

The date of birth was recorded to the nearest week by the data custodian in accordance with Information Governance policy. Covariates derived from the WECC datasets were academic season of birth, sex, gestation, maternal age, parity, multiple births, congenital anomaly (coded as none, major or minor), whether the child was admitted in the perinatal period (<7 days), whether the child was born by caesarean section, was small for gestation (below the 10^th^ centile of birthweight adjusted for gestation and sex), maternal smoking during pregnancy, and breastfeeding at birth or at 6–8 weeks of age. Data on maternal smoking during pregnancy and breastfeeding are collected by midwives and health visitors respectively and entered on to local electronic child health systems that are used for clinical management and administrative purposes.

#### Socio-demographic variables

We used data from the NPD for each child’s on eligibility for free school meals, as a proxy for socioeconomic status before age 7 years (defined as a family receiving income-based benefits, e.g. Income Support or Child Tax Credit, in 2007 classified as families with an income below around £15,000). To measure the social deprivation of the area of residence we used the Welsh Index of Multiple Deprivation (WIMD) 2005 at lower super output area (LSOA) level, equivalent to a postcode area with a minimum of 1000 houses and mean 1500 houses [29]. We divided the 1896 LSOAs in Wales into five quintiles of approximately equal counts. WIMD 2005 uses geographical data from the 2001 census, and 8 domains of relative deprivation (income, employment, health, education, community safety, housing, physical environment and geographical access to services). This measure was chosen to include the broadest group of measures at the mid-point of the 1998–2005 cohort follow-up. Guidance on the use of WIMD [30] states that valid comparisons can be made between deprivation deciles over time, and shows that reference periods for some of the domain measures stretch back over the last 10 years.

Previous research indicated that neighbourhood deprivation was an important determinant of educational attainment. In order to assess the influence of moving house between neighbourhoods, we extracted each child’s LSOA at birth and also their LSOA at the usual age of taking KS1, at age 6–7 years. Each of these LSOAs was classified as being above or below the median of the WIMD score and four categories were defined, based on the initial WIMD score and the score at the time of taking KS1. Each child was placed in one of these categories: remained not deprived, moved from not deprived to deprived, moved from deprived to not deprived, or remained deprived. We used only the deprivation quintile at birth and the last deprivation quintile before KS1 for simplicity (some children had moved up to 30 times). We also assigned each child their ONS urban/rural settlement type classification of their neighbourhood at birth [31] as this was a factor associated with educational attainment in previous research [32].

#### School-level variables

School-level data available from the Local Education Authorities were anonymously linked to our cohort. We divided school size into bands of ≤100 pupils, 101–200, 201–300 and >300. We calculated a school-based measure of deprivation for each school catchment area using the annual percentage of children eligible for free school meal in each school and creating an average for each school over 2006 to 2008. This was categorised into ≤10%, 11–20, 21–30, 31–40, >40%.

We also derived a variable for the number of school moves for each child during the reception year or school years 1 and 2 based on change in the school reference code assigned to the child and recorded in the PLASC in January of each year, and additionally in May in Key Stage years.

### Statistical analysis

Using MLwiN software [33] we fitted multilevel logistic regression models, with not attaining the expected level in KS1 as the outcome. We specified the hierarchical structure of children nested within schools within LEAs. First we fitted the null random-effects model, quantifying the variation in the risk of poor educational attainment with random intercept terms for schools and LEAs.

In model 1 we added terms for the emergency inpatient hospital admission variables, estimating the unadjusted risk of poor educational attainment associated with hospital admission. In model 2 we fully adjusted for all the individual level variables and estimated the associations between the outcome and hospital admission, pregnancy factors, birth characteristics and socio-economic status (using the free school meals variable). We repeated the model using each of the cause-specific hospital admission variables to prevent possible effects of collinearity between hospital variables. Finally, in model 3 we modelled added terms for the area-level and school-level variables to adjust for associations between these and educational attainment.

Several pregnancy and perinatal variables had missing values for small numbers of children but there was a large proportion of missing data for breastfeeding (50.0%) and maternal smoking (49.0%) during pregnancy. Descriptive analyses of these missing data by year and Unitary Authority (county or county borough councils) showed a fairly uniform pattern of missingness across Wales, and for reasons likely to be unconnected to true breastfeeding or smoking status. In fact, the highest percentages of recording were found to exist where incentives to collect data were known about, and we judged such administrative reasons for missingness to be ignorable [34]. Where these variables are recorded, there is of course a risk of social desirability bias, whereby maternal smoking in pregnancy may be underestimated or breastfeeding overestimated. The effect of such measurement error, as usual, is to attenuate real differences between groups.

The sample size with observed data for these two variables was sufficient to estimate the effect of their association with the outcome and their 95% credible intervals.

We applied multiple imputation using chained equations [35], with all variables included in the imputation models to generate five imputed datasets, and derived pooled estimates using Rubin’s rules [36]. Parameters were estimated for the fully adjusted models using Markov chain Monte Carlo (MCMC) methods [37], with second order penalised quasi-likelihood estimates as starting values. We ran chains of length 30,000, based on the run-length diagnostics, and derived 95^th^ credible intervals for each parameter.

For binary outcome models, variation attributable to different hierarchical levels in a multilevel model are only approximated by intraclass correlation coefficients, and explained variation R^2^ calculations are problematic. Therefore model variation has additionally been described using the median odds ratio (MOR) for each hierarchical level and allows direct comparison to fixed effect ORs on the outcome. The MOR quantifies the magnitude of the effect of clustering and is described as the median odds ratio from the repeated sampling at random of two subjects with the same covariates from different clusters (differences are quantified entirely by cluster-specific random effects) [38].

### Ethics and research governance

The study design uses anonymised data and therefore the need for ethical approval and participant consent was waived by the approving Institutional Review Board, UK National Health Service Research Ethics Committee. The SAIL independent Information Governance Review Panel, which contains members from the UK National Health Service Research Ethics Committee, experts in information governance and members of the public, approved the study.

## Results

The cohort comprised 64 934 children in 1463 schools, nested within 22 LEAs. Overall, 4680 (7.2%) did not achieve the expected educational level. About half of the children in the cohort (48.4%) had an emergency inpatient hospital admission (Table 2). The majority of these occurred during the first year of life. The fully adjusted odds ratio for at least one emergency inpatient hospital admission was 1.12 (95^th^ credible interval 1.05, 1.20), with an admission due to an injury or external cause or being admitted in infancy having the strongest associations with the educational outcome (Table 3). The number of bed days in the pre-school age group was associated with an increased risk of any emergency inpatient admission, but the number of bed days during school-age was not significant in the model.

**Table 2:** Emergency inpatient admissions, birth and socio-demographic characteristics, and school factors by not attaining Key Stage 1 in education at age 6–7 years.

Characteristic		Children in Cohort N = 64,934 n (%)	Not attained KS1 n = 4,680 n (%)
**Emergency inpatient admissions**			
Any admission	No	33475 (51.6)	2143 (6.4)
	Yes	31459 (48.4)	2537 (8.1)
Any respiratory admission	No	52152 (80.3)	3593 (6.0)
	Yes	12782 (19.7)	1087 (8.5)
Any admission for external causes	No	59325 (91.4)	4144 (7.0)
	Yes	5609 (8.6)	536 (9.6)
Any gastro-intestinal admission	No	61042 (94.0)	4362 (7.1)
	Yes	3892 (6.0)	318 (8.2)
Any other admission	No	44782 (69.0)	3034 (6.8)
	Yes	20152 (31.0)	1646 (8.2)
Number of admissions	0	33475 (51.6)	2143 (6.4)
	1	17721 (22.3)	1331 (7.5)
	2	7420 (11.4)	620 (8.4)
	≥3	6318 (9.7)	586 (9.3)
Age at first admission	No admission	33475 (51.6)	2143 (6.4)
	<1	15778 (24.3)	1355 (8.6)
	1–4	12429 (19.1)	924 (7.5)
	>4	3256 (5.0)	248 (7.6)
Number of bed-days pre-school	10**	1.99(SD 5.57)^	
Number of bed-days at school	10**	0.25 (SD 1.68)^	
**Birth characteristics**			
Gender	Female	30239 (46.6)	2579 (8.5)
	Male	34695 (53.4)	2101 (6.1)
Gestational age	≤32	678 (1.0)	65 (9.6)
	33–36	3303 (5.1)	284 (8.6)
	37–39	24051 (37.0)	1821 (7.6)
	40–42	36902 (56.8)	2510 (6.8)
Maternal age	≤19	5754 (8.9)	702 (12.2)
	20–24	12124 (18.7)	1189 (9.8)
	25–29	19646 (30.3)	1263 (6.4)
	30–34	17631 (27.2)	930 (5.3)
	35–39	7024 (10.8)	399 (5.7)
	≥40	1122 (1.7)	74 (6.6)
	No answer	1633 (2.5)	123 (7.5)
Parity	0	28354 (43.7)	1731 (6.1)
	≥1	36477 (56.2)	2943 (8.1)
	No answer	103 (0.2)	6 (5.8)
Multiple births	No	63312 (97.5)	4576 (7.2)
	Yes	1622 (2.5)	1104 (6.4)
Perinatal/neonatal inpatient admissionǂ	No	60343 (92.9)	4309 (17.1)
	Yes	4591 (7.1)	371 (8.1)
Congenital anomaly	No	60885 (93.8)	4345 (7.1)
	Minor	1890 (2.9)	146 (7.7)
	Major	2159 (3.3)	189 (8.8)
Caesarean section	No	49861 (76.8)	3644 (7.3)
	Yes	13443 (20.7)	513 (6.8)
	No answer	1630 (2.5)	123 (7.5)
Small for gestational age	No	59014 (90.9)	4067 (6.9)
	Yes	5920 (9.1)	613 (10.4)
Breast feeding	No	14548 (22.4)	1329 (9.1)
	Yes	17925 (27.6)	1098 (6.1)
	No answer	32461 (50.0)	2253 (6.9)
Smoking	No	10628 (16.4)	620 (5.8)
	Yes	2800 (4.3)	361 (12.9)
	Missing	51506 (49.3)	3699 (7.2)
Academic season of birth	Sept-Dec	18312 (28.2)	896 (4.9)
	Jan-Apr	23724 (36.5)	1683 (7.1)
	May-Aug	22898 (35.3)	2101 (9.2)
**Socio-demographic characteristics**			
Free School meals	No	55587 (85.6)	3207 (5.8)
	Yes	9347 (14.4)	1473 (15.8)
Townsend deprivation quintile at birth / first 4 months	1 least deprived	12392 (19.1)	461 (3.7)
	2	11889 (18.3)	653 (5.5)
	3	12680 (19.5)	867 (6.8)
	4	13005 (20.0)	985 (7.6)
	5 most deprived	14968 (23.1)	1714 (11.5)
Deprivation status change, using the Townsend score at birth / first 4 months and at taking KS1	Stayed low and least deprived (below median)	26500 (40.8)	1119 (4.5)
	Low to high (below to above median)	4321 (6.7)	342 (7.9)
	High to low (above to below median)	6316 (9.7)	424 (6.7)
	Stayed high and most deprived (above median)	27797 (42.8)	2715 (9.8)
Living environment in birth / first 4 months	Urban (>10K pop)	44879 (69.1)	3300 (7.4)
	Town & Fringe	10795 (16.6)	759 (7.0)
	Village, hamlet & isolated dwellings	9260 (14.3)	621 (6.7)
**School factors**			
Number of school moves	0	58795 (90.5)	3814 (6.5)
	1	5787 (8.9)	779 (13.5)
	2+	352 (0.5)	87 (24.7)
Average school size between 2006 and 2008	≤100	6459 (9.9)	544 (8.4)
	101–200	19983 (30.8)	1564 (7.8)
	201–300	19147 (29.5)	1261 (6.6)
	>300	19345 (29.8)	1311 (6.8)
Average percentage of children eligible for free school meals in school during 2006–8	≤10	26672 (41.1)	1336 (5.0)
	10–20	21212 (32.7)	1420 (6.7)
	20–30	9332 (14.4)	839 (9.0)
	30–40	4980 (7.7)	565 (11.3)
	>40	2738 (4.2)	520 (19.0)

An emergency inpatient admission is a continuous inpatient spell of finished consultant episodes and includes transfers between hospitals using the Dr Foster superspells method [59]. Type of admission is derived from the first 3 diagnosis codes in the first consultation episode for each emergency inpatient admission.

^ Mean(Standard Deviation).

ǂ emergency or elective.

** The OR’s shown here correspond to a difference per 10 days bed-days.

**Table 3:** Multilevel logistic regression for not attaining Key Stage 1 at age 6–7 years and emergency inpatient hospital admissions (emergency hospital admission variables were entered individually into adjusted models), N = 64934.

Characteristic		Unadjusted OR (95% CrI)	Model 2 with partially adjusted OR* (95% CrI)	Model 3 with fully adjusted OR** (95% CrI)
Any admission	No	1	1	1
	Yes	1.23 (1.15, 1.31)	1.14 (1.07, 1.22)	1.12 (1.05,1.20)
Any respiratory admission	No	1	1	1
	Yes	1.23 (1.14, 1.33)	1.13 (1.04,1.22)	1.10 (1.01, 1.20)
Any admission for external causes	No	1	1	1
	Yes	1.31 (1.18, 1.45)	1.20 (1.08, 1.33)	1.19 (1.07,1.32)
Any gastro-intestinal admission	No	1	1	1
	Yes	1.09 (0.98, 1.21)	1.03 (0.90,1.18)	0.99 (0.87, 1.14)
Any other admission	No	1	1	1
	Yes	1.18 (1.10, 1.26)	1.11 (1.03,1.19)	1.09 (1.01, 1.17)
Number of admissions	0	1	1	1
	1	1.17 (1.08, 1.26)	1.12 (1.03, 1.21)	1.11 (1.02, 1.20)
	2	1.26 (1.14, 1.40)	1.18 (1.06, 1.31)	1.14 (1.02, 1.26)
	≥3	1.37 (1.3, 1.52)	1.17 (1.05, 1.31)	1.13 (1.01, 1.26)
Age at first admission	No admission	1	1	1
	<1	1.30 (1.20,1.41)	1.16 (1.06,1.25)	1.31 (1.04, 1.22)
	1–4	1.15 (1.06,1.26)	1.12 (1.03,1.22)	1.11 (1.01, 1.21)
	>4	1.18 (1.02,1.37)	1.16 (1.00, 1.35)	1.15 (0.99, 1.34)
Number of bed-days pre-school	10***	1.13 (1.08,1.18)	1.14 (1.07,1.21)	1.12 (1.05,1.19)
Number of bed-days at school	10***	1.09 (0.96, 1.25)	1.06 (0.91, 1.22)	1.05 (0.90, 1.22)

An emergency inpatient admission is a continuous inpatient spell of finished consultant episodes and includes transfers between hospitals using the Dr Foster superspells method [59]. Type of admission is derived from the first 3 diagnosis codes in the first consultation episode for each emergency inpatient admission.

* Partially adjusted ORs are adjusted for gender, gestational age, maternal age, parity, multiple births at childbirth, congenital anomaly, perinatal/neonatal inpatient admission, caesarean section, small for gestational age (<10^th^ centile), breastfeeding, maternal smoking in first trimester, academic season of birth, free school meal eligible (S1 Table).

** Fully adjusted ORs are adjusted for all the variables in the partially adjusted model and area-level and school-level variables (Townsend deprivation quintile at birth, deprivation status change between birth and KS1, living environment at birth, number of school moves, average school size, average percentage of children eligible for free school meal at school; S1 and S2 Tables).

*** ORs shown correspond to a difference per 10 bed-days. OR = Odds ratio; CrI = Credible interval.

The proportion not attaining KS1 was significantly higher in a number of groups, most notably in boys, those born extremely preterm, maternal age under 20, not being a first child, being small for gestational age, being eligible for free school meals, being born between January and August (meaning they were young for their school year) (Table 2), moving school, living in areas of higher social deprivation and attending a school with a high percentage of pupils eligible for free school meals.

The associations between emergency hospital inpatient admission variables and educational attainment were weaker than the observed effects associated with pregnancy, perinatal and social and school environment variables (S1 and S2 Tables) but there remained an independent association between emergency inpatient hospital admissions and educational attainment in the adjusted models.

In the unadjusted multilevel null model for any emergency inpatient hospital admission, 2.3% of the unexplained variation in the educational outcome was attributable to the LEAs, 25.4% to schools and 72.3% to individual variation. The model fit statistic measured with the Bayesian deviance information criterion (DIC) was 29349.52 on average. In the fully adjusted models these percentages changed little, to 2.7%, 22.1% and 75.2%, respectively; average Bayesian DIC 27581.45. The variance attributable to the hierarchical levels on the outcome and fit statistics for the null model, and models 1 to 3 are reported in Table 4.

**Table 4:** Hierarchical variation and fit in multilevel logistic regression for any emergency inpatient hospital admission and not attaining Key Stage 1 at age 6–7 years, N = 64934.

	Null model	Model 1 with any hospital admission	Model 2 with any hospital admission and individual level characteristics	Model 3 with any hospital admission, individual level, area-level and school-level characteristics
**Unexplained variance (SE)**				
Child	3.29	3.29	3.29	3.29
School	1.119 (0.073)	1.113 (0.073)	0.972 (0.066)	0.934 (0.065)
Local Education Authority	0.101 (0.047)	0.106 (0.050)	0.092 (0.042)	0.092 (0.042)
**Percentage of unexplained variance: intraclass correlation (ICC)**				
Child	72.3	71.6	74.5	75.2
School	25.4	25.3	22.8	22.1
Local Education Authority	2.3	3.1	2.7	2.7
**Explained variation (%)**				
School	Reference	0.5	13.1	16.5
Local Education Authority	Reference	4.7	8.9	8.9
**Median odds ratio (MOR)**				
School	2.74	2.74	2.56	2.51
Local Education Authority	1.35	1.36	1.34	1.34
**Model fit statistic**				
Bayesian deviation information criterion (DIC)	29349.52	29313.83	28015.08	27581.45

The analyses were repeated for the outcome measures of not attaining the expected level in each of language, mathematics and science, separately. For language 4.6% of children did not achieve the expected standard, in mathematics 3.9% and in science 2.8%. The ORs associated with any emergency inpatient hospital admission were 1.07 (0.98, 1.16) for language, 1.16 (1.06, 1.27) for mathematics and 1.06 (0.95, 1.19) for science, suggesting mathematics results were a key factor in the association between overall KS1 results and emergency inpatient hospital admission. For science, considerably more variation (31%) was attributable to differences between schools; for maths and language the results were comparable to those for the overall assessment.

## Discussion

In this electronic birth cohort we have shown that emergency inpatient admission to hospital in the first seven years of life is associated with poor educational attainment in standardised teacher assessments at age seven years. This effect was greater for admissions due to injuries and external causes and for admissions in infancy, and increased with more bed days spent in hospital during the pre-school period. These observed increased risks are probably a conservative estimate as our analysis excluded children with special educational needs, some of whom may have had a higher number of emergency inpatient hospital admissions [38] due to more specialist care required or complications with their other conditions [39].

**Fig 2:**
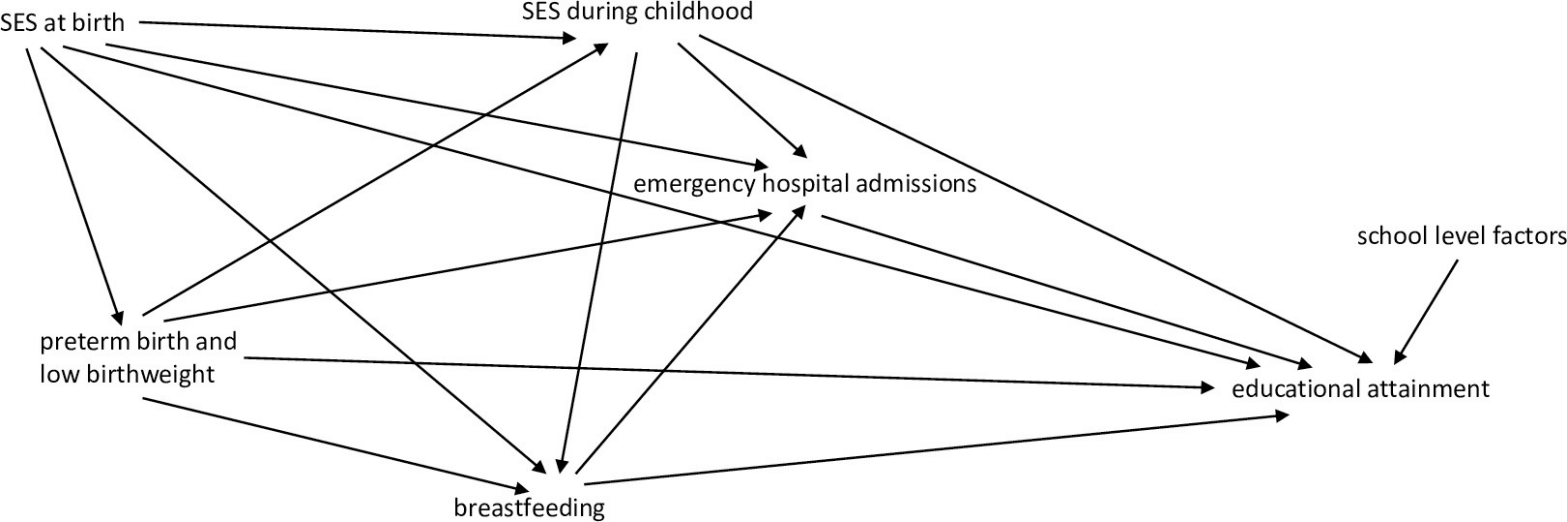
Visual diagram of causal relationships for selection of confounder variables. SES = Socio-economic status.

The determinants of educational outcome in children are multifactorial, with complex interactions between these factors (see Fig 2). Most previous studies have focused on the association between education outcomes and various combinations of factors, such as health status measured at birth (gestational age [6,9–18], birth weight [6–8] and Apgar score [40]), socio-economic and school-level factors. Few studies have examined how health during childhood, which is partially influenced by birth health status, impacts on educational outcomes. Hospital admissions, particularly for respiratory diseases, which are more common in infants born late preterm and early term [23,41], may have a role on the causal pathway in the well-documented relationship between preterm birth and lower educational outcome. Poor health due to chronic conditions can affect a child’s daily activities, social interactions and school attendance, either due to the illness itself or the treatment associated with it [42–47]. The biological insults resulting in a child’s health status may influence the neural connections required for optimal development, affecting concentration, memory and general cognitive ability. Most previous studies have either measured health during childhood using self-reports of general health condition, duration of hospital admissions or studied disease specific cohorts. Few studies have examined the impact of acute or transient conditions requiring hospital admission (such as infections and injuries) on education outcomes.

Our review of the literature identified seven previous studies that specifically examined the impact of unplanned hospital inpatient admissions on educational outcome. [1,2,48–52] Consistent with our findings, O’Brien Caughy’s analysis of secondary data in the U.S.A. showed that inpatient hospital admissions during the first year of life was associated with lower scores on reading recognition, partially explained by differences in levels of maternal education and the home environment [1]. Kull et al tested the associations between child development at age five years and indicators of physical health during childhood (including acute conditions and unplanned hospitalisations) in a large national birth cohort of children in Boston U.S.A. [2]. Their analyses showed that multiple aspects of child health, specifically, neonatal risks due prematurity and birth weight, poor general health and unplanned hospitalisation for asthma or respiratory infection independently predicted lower cognitive and learning skills and lower pro-social skills [2]. Bell et al examined the association between chronic illness and school readiness using linked administrative population based healthcare data in Western Australia, adjusted for child, parent and community socio-demographic variables. They reported that children with a chronic illness (including conditions such as chronic otitis media), measured using admission to hospital or emergency department attendance, had an increased risk of developmental vulnerability and reduced school readiness [51]. Kohler-Forsberg et al showed that hospital inpatient admissions for infections were associated with subsequent decreased cognitive ability and lower educational outcomes at age 15–16 years [50]. This may be due to missed school days, or the confounding effects of socio-economic status, which are also associated with an increased risk of hospital admissions. There is also increased recognition that a wide range of infections may impact on brain function via cytokines and/or inflammatory markers [53]. Infections are the most common cause of inpatient hospital admissions, particularly in younger children. Our finding of a larger effect size for the association between inpatient hospital admissions during the first year of life and lower education outcomes at age 6–7 years may reflect the effects of infections or other insults on the developing brain. Pre-term birth is often associated with perinatal complications requiring hospital inpatient admission and can also lead to compromise of the central nervous system increasing the risk of poor cognitive development and subsequent lower educational outcomes. We found an independent effect of emergency hospital inpatient admissions on education outcomes, over and above the increased risks associated with pre-term births. This suggests that unplanned hospital inpatient admissions, particularly during the first year of life, may be an early indicator of children who require early intervention and additional support to achieve their academic potential.

Three of the seven studies we identified focused specifically on hospital admissions due to injuries. Sesko et al showed that paediatric orthopaedic injuries and their treatment contribute to school absence, suggesting the need for school policies that accommodate the needs of the injured child [42]. Of the children who received hospital treatment in this study, 47% were unable to return immediately to school, and on average these children had up to 40 days absent from school [42]. Evidence from large-scale robust studies in the USA have shown that school absence in the elementary years is associated with poor educational outcomes in reading and mathematics [54–55]. Gabbe et al quantified the impact of inpatient admission for head injury on academic performance in the Wales Electronic Cohort for Children (WECC), showing that children who had sustained an intracranial injury had an increased risk of not attaining the expected level of achievement at age 6–7 years, using the same outcome measure that we used in our analysis [48]. This could be a direct effect of temporary or permanent brain damage or indirect effect of time missed from school. Azzam et al examined the influence of burn injuries in national standardised curriculum-based school tests among children aged 8–14 years in Australia. They found that the most childhood burn injuries occur during the pre-school years, and children who were hospitalised had lower performance on academic assessments, suggesting that rehabilitation programmes for children with burn injuries should also include educational support for children [52].

To measure the time lost from school during an emergency inpatient hospital admission we assessed the number of bed days for each child in the analysis and found a significant effect for this in the pre-school period, but not during the three years of school in the KS1 period. This may potentially reflect the fact that neonatal and other chronic conditions that can have significant consequences for development during the life-course are most prevalent during the pre-school years (before age 5 years), whereas after age 5 injuries and external causes are more prevalent causes of morbidity [56]. However, we had no available information on days of absence post-discharge, nor on school absence generally. Not being able to include the full school absence from hospital admission in the analysis would tend to bias our results to the null, suggesting the possibility that the true effect of emergency inpatient hospital admission on KS1 attainment is stronger than we estimated.

Unplanned hospital inpatient admissions are easily identifiable events that are potentially disruptive to the lives of children and their families. Our results add to the body of evidence from previous studies that children who have been admitted to hospital before starting school, particularly in the presence of other socio-economic indicators may represent a group that need additional support to ensure they reach their academic potential. Injuries and external causes are a major cause of morbidity in school-aged children. Our finding of increased risk of lower educational attainment associated with injuries and external causes, therefore suggests that rehabilitation programmes for these children should also address their educational needs and effective injury prevention strategies are needed for school children, to reduce the incidence of these types of admissions and their consequences for children.

The effects associated with unplanned hospital inpatient admissions in our analysis are rather less than those for many perinatal factors, particularly being extremely premature, being small for gestational age, and for socio-economic measures, including eligibility for free school meals, living in deprived areas and attending a school with a high proportion of pupils eligible for free school meals. It was unsurprising that moving schools is associated with lower levels of attainment and it is striking, in line with previous studies [57], that there is a strong association with being born late in the school year, with such a child being younger on school entry and also when being assessed. We examined the effect of school size and found that smaller schools were associated with an increased risk of not attaining KS1. Previous research has shown that while smaller schools may be favoured by parents for children with special educational needs (who may not be receiving SEN provision), larger schools may have more services to help all learners. However, lower reading scores were also observed for children from more deprived areas as school size became larger [58].

Although the majority of the variation in the educational outcome was explained by differences between children, it is an important finding that over 20% of the variation in educational attainment may be explained by factors that are correlated within a school. In contrast, only 2% of the variation was attributable to differences between LEAs.

This study has the important strength of record-linking longitudinal perinatal data on all 64, 934 children born in Wales over three school years who satisfied the eligibility criteria. The cohort included complete data on educational attainment and inpatient hospital admissions, and a wide range of clinical and socioeconomic factors. We were able to link children with their schools and investigate the potentially important school-level influences on educational achievement, as well as the effects of changes in small-area deprivation from moving house.

There are a number of limitations primarily concerned with missing data or data quality. Our measure of health status during childhood was emergency inpatient hospital admissions. The majority of these admissions are due to respiratory and gastrointestinal infections in younger children, followed by injuries and external causes in older children. In addition to clinical need, hospital admissions may also reflect supply side factors such as availability and ability to access primary care, and other socially patterned factors. However, there was an increased risk of lower attainment at KS1 even after we adjusted for all available measures of socio-economic status and social deprivation in the model. Children attending independent schools were not included as the education datasets were restricted to children in schools maintained by the LEA. It is possible that this introduced some selection bias but fewer than 2% of primary school children attend independent schools in Wales and so any resulting bias is unlikely to be large. Children with special educational needs were also excluded because they are considerably less likely to achieve the expected standard at KS1 and their inclusion would have introduced considerable heterogeneity. However, we acknowledge that head injuries can lead to learning difficulties, and hence this exclusion could have biased our estimate for the effect of head injuries towards the null.

Data on the cause of an emergency inpatient hospital admission depends on the accuracy of coding. We reviewed the codes used by NHS coders and found little variation in the codes for similar causes of admission, so any misclassification was likely to be minimal and unlikely to have a major effect on the results.

Children without a valid birthweight or gestational age were excluded but other data were imputed using a standard method of chained equations. The percentage of missing data was substantial only for breastfeeding and smoking, due to organisational and administrative differences in data collation between hospitals in Wales, suggesting that these data can be reasonably assumed to be missing at random. However, the subset for which data were available was large enough to fit an imputation model for these covariates with sufficient precision, and the variation of the estimates of associations between KS1 attainment and hospital admissions varied very little between imputations, suggesting the procedure was robust. We chose to impute these variables because they are key factors that are socially patterned and known determinants of child health status.

It is also possible that the relationship between emergency hospital inpatient admission and educational attainment is confounded by unmeasured factors associated with socio-economic status. We adjusted for as many of these as was possible to measure in this analysis of administrative health and education datasets, but there may be other measures of socio-economic status such as maternal education level or home environment we were unable to control for. However we were able to adjust for eligibility for free school meals, defined by eligibility for means-tested income support benefit, as a measure of family socio-economic status, and small-area deprivation measured by the Welsh index of multiple deprivation.

## Conclusion

Emergency inpatient hospital admission during childhood, particularly during infancy or for injuries and external causes is associated with lower educational attainment at age seven years, even after allowing for the effects of pregnancy factors such as gestational age and birthweight, and school-level factors. These findings suggest that emergency inpatient hospital admissions can be used to measure an additional effect on educational attainment beyond birth health status, several measures of environment, deprivation and school factors. These children may need a review of their health and support networks, possible additional learning support in school to achieve their potential, and the results strengthen the case for more effective injury prevention strategies.

## Supporting information

S1 Table Birth and individual covariates from multilevel logistic regression for not attaining Key Stage 1 at age 6–7 years and emergency inpatient hospital admissions in Table 3, N = 64,934. (TIF)

S2 Table Area-level deprivation and school-level covariates from multilevel logistic regression for not attaining Key Stage 1 at age 6–7 years and emergency inpatient hospital admissions in Table 3, N = 64,934. (TIF)

## Abbreviations

ALF_E: Anonymised Linking Field
AWPS: All Wales Perinatal Survey
CARIS: Congenital Anomaly Register and Information Service
KS1: Key Stage 1
LEA: Local Education Authority
LSOA: Lower Super Output Area
MCMC: Markov chain Monte Carlo
NCCHD: National Community Child Health Database
NPD: National Pupil Database
PEDW: Patient Episode Database for Wales
ONS: Office for National Statistics
PLASC: Pupil Level Annual School Census
SAIL: Secure Anonymised Information Linkage
SEN: Special Education Needs
WDS: Welsh Demographic Service
WECC: Wales Electronic Cohort for Children
WIMD: Welsh Index of Multiple Deprivation
