# Supplementary figures and images for: The role of health and social factors in education outcome: A record-linked electronic birth cohort analysis
Source: PLoS One. 2019 Aug 9;14(8):e0220771. doi: 10.1371/journal.pone.0220771 (PMC6688802; doi:10.1371/journal.pone.0220771)

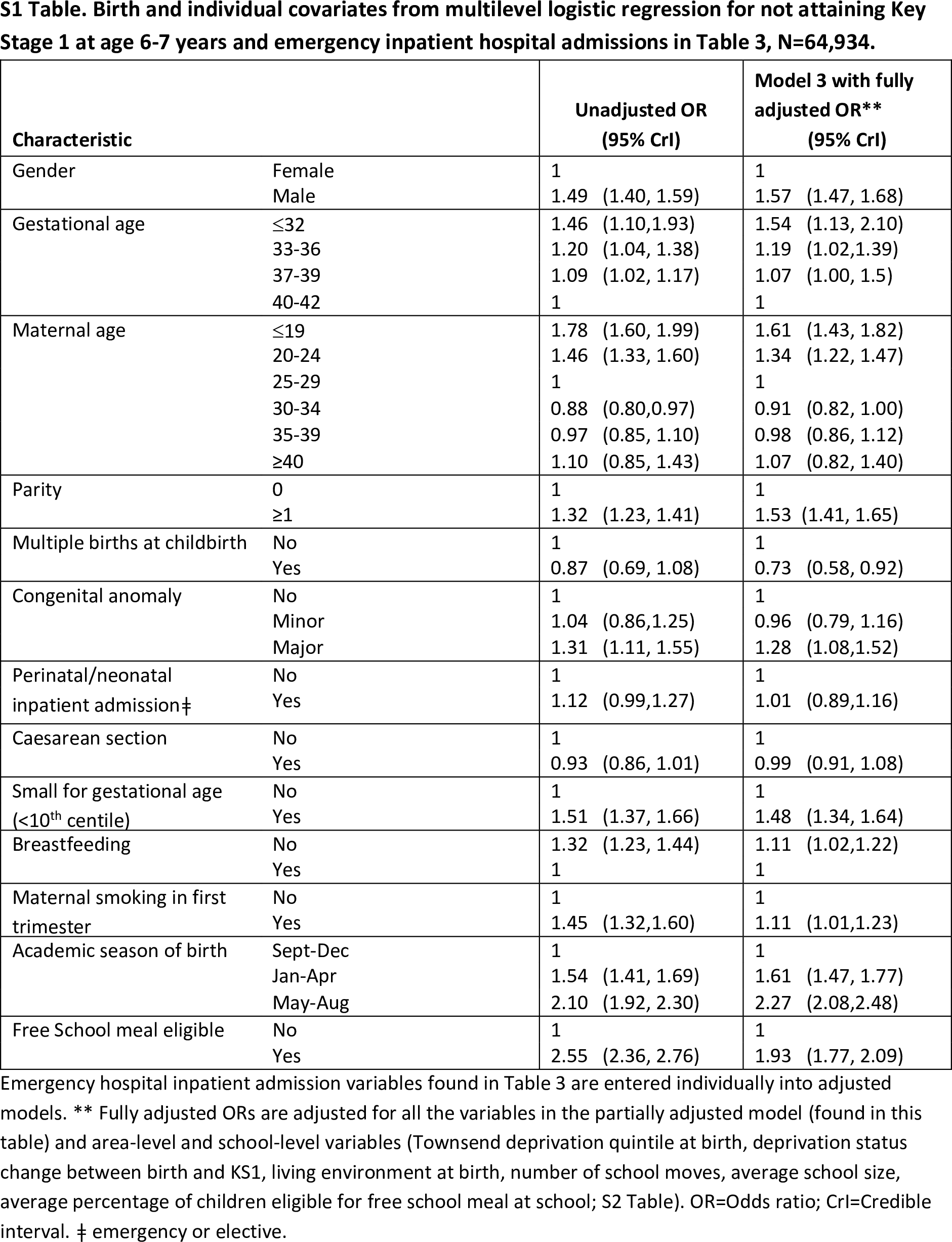

Supplement: S1 Table — (TIF) [file pone.0220771.s001.tif]

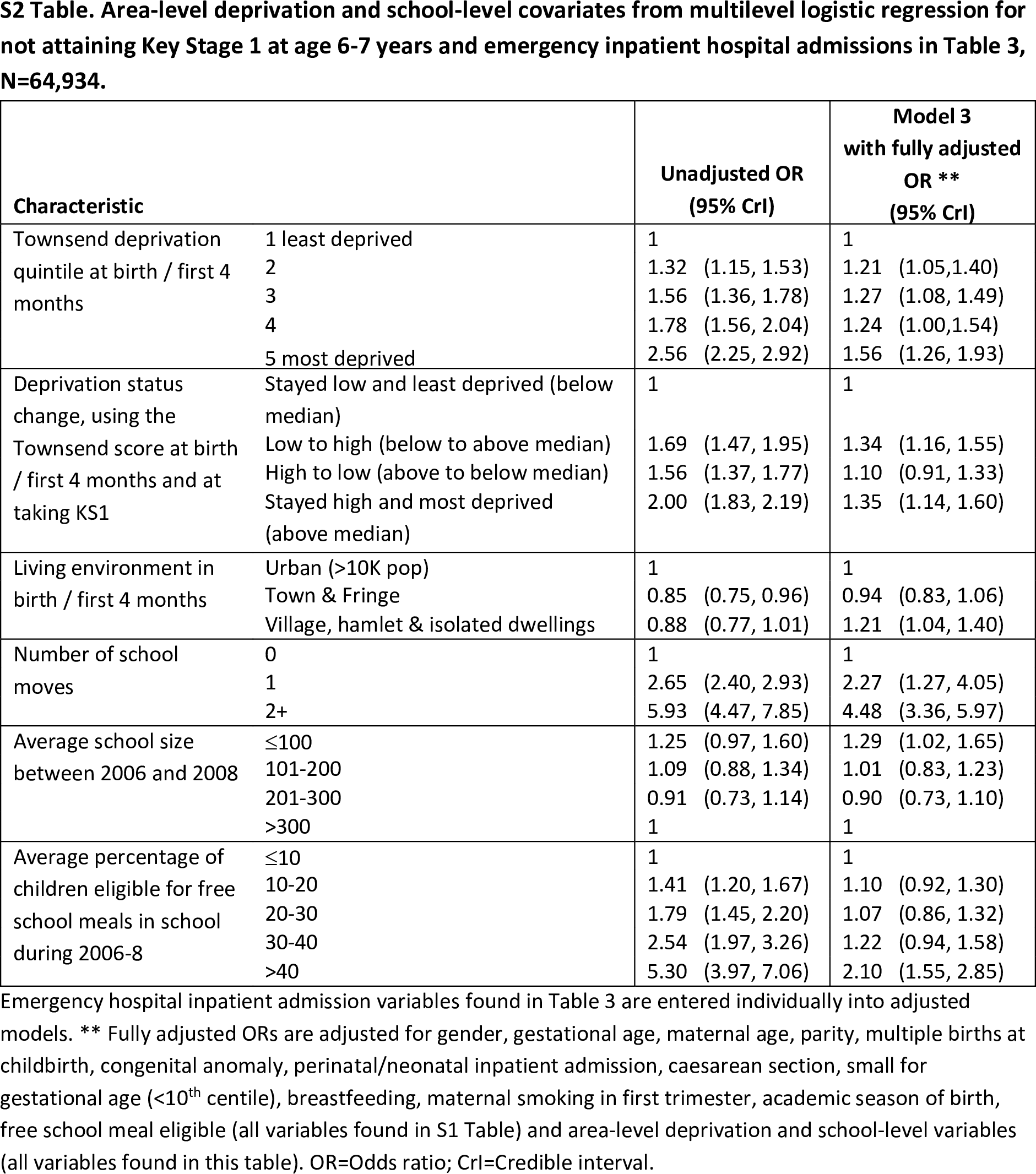

Supplement: S2 Table — (TIF) [file pone.0220771.s002.tif]
